# Multiple Layers of Phospho-Regulation Coordinate Metabolism and the Cell Cycle in Budding Yeast

**DOI:** 10.3389/fcell.2019.00338

**Published:** 2019-12-17

**Authors:** Lichao Zhang, Sebastian Winkler, Fabian P. Schlottmann, Oliver Kohlbacher, Josh E. Elias, Jan M. Skotheim, Jennifer C. Ewald

**Affiliations:** ^1^Department of Chemical and Systems Biology, Stanford University, Stanford, CA, United States; ^2^Applied Bioinformatics, Department of Computer Science, University of Tübingen, Tübingen, Germany; ^3^Molecular Cell Biology, Interfaculty Institute of Cell Biology, University of Tübingen, Tübingen, Germany; ^4^Institute for Translational Bioinformatics, University Hospital Tübingen, Tübingen, Germany; ^5^Institute for Bioinformatics and Medical Informatics, University of Tübingen, Tübingen, Germany; ^6^Quantitative Biology Center, University of Tübingen, Tübingen, Germany; ^7^Biomolecular Interactions, Max Planck Institute for Developmental Biology, Tübingen, Germany; ^8^Department of Biology, Stanford University, Stanford, CA, United States

**Keywords:** cell division cycle, metabolism, proliferation, phosphoproteomics, *Saccharomyces cerevisiae*, CDK, PKA, PP1

## Abstract

The coordination of metabolism and growth with cell division is crucial for proliferation. While it has long been known that cell metabolism regulates the cell division cycle, it is becoming increasingly clear that the cell division cycle also regulates metabolism. In budding yeast, we previously showed that over half of all measured metabolites change concentration through the cell cycle indicating that metabolic fluxes are extensively regulated during cell cycle progression. However, how this regulation is achieved still remains poorly understood. Since both the cell cycle and metabolism are regulated to a large extent by protein phosphorylation, we here decided to measure the phosphoproteome through the budding yeast cell cycle. Specifically, we chose a cell cycle synchronization strategy that avoids stress and nutrient-related perturbations of metabolism, and we grew the yeast on ethanol minimal medium to force cells to utilize their full biosynthetic repertoire. Using a tandem-mass-tagging approach, we found over 200 sites on metabolic enzymes and transporters to be phospho-regulated. These sites were distributed among many pathways including carbohydrate catabolism, lipid metabolism, and amino acid synthesis and therefore likely contribute to changing metabolic fluxes through the cell cycle. Among all one thousand sites whose phosphorylation increases through the cell cycle, the CDK consensus motif and an arginine-directed motif were highly enriched. This arginine-directed R-R-x-S motif is associated with protein-kinase A, which regulates metabolism and promotes growth. Finally, we also found over one thousand sites that are dephosphorylated through the G1/S transition. We speculate that the phosphatase Glc7/PP1, known to regulate both the cell cycle and carbon metabolism, may play an important role because its regulatory subunits are phospho-regulated in our data. In summary, our results identify extensive cell cycle dependent phosphorylation and dephosphorylation of metabolic enzymes and suggest multiple mechanisms through which the cell division cycle regulates metabolic signaling pathways to temporally coordinate biosynthesis with distinct phases of the cell division cycle.

## Introduction

For cells to proliferate, they need to coordinate cell growth driven by metabolism with the cell division cycle, which ensures that DNA and other crucial cellular components are duplicated and divided between two daughter cells. In budding yeast, it was viewed that cell metabolism and growth proceed largely independently of the cell cycle. This assumption comes from the observation that mutants arrested in distinct phases of the cell cycle continued to grow and became extremely large and irregularly shaped (Hartwell et al., 1974; Johnston et al., 1977; Pringle and Hartwell, 1981). This showed clearly that a cell cycle arrest does not stop metabolism and mass accumulation, which led to the text book model that in budding yeast growth controls division, but not vice versa (Morgan, 2007).

While the hierarchy of metabolism driving the cell cycle was long the consensus, many studies over this past decade have challenged this view. It now seems that metabolism, growth, and division are tightly and multi-directionally coordinated in all eukaryotes including yeast (Goranov and Amon, 2010; Papagiannakis et al., 2017; Ewald, 2018; Ozsezen et al., 2019). Indeed, several core cell cycle regulators also target metabolic pathways and thereby control metabolism and growth: The most central cell cycle regulator, the cyclin-dependent kinase (CDK), has been found to directly target proteins in carbohydrate and energy metabolism in yeast (Ewald et al., 2016; Zhao et al., 2016), flies (Icreverzi et al., 2012) and mammals (Galbraith et al., 2017; Wang et al., 2017; reviewed in Solaki and Ewald, 2018). Moreover, in addition to its role in mitosis, the polo kinase routes fluxes through the pentose-phosphate pathway by phosphorylating glucose-6-phosphate dehydrogenase in human cancer cell lines (Ma et al., 2017), and the cell cycle regulated ubiquitin ligase APC/C (anaphase promoting complex) regulates glucose metabolism in HeLa cells (Tudzarova et al., 2011). However, while specific examples of cell cycle regulators controlling metabolic pathways are accumulating, the global scope of metabolic regulation during the cell cycle is still largely unexplored.

The global regulation of metabolic processes during cell cycle progression is likely to be vast because 50% of the measured metabolites in budding yeast change concentration significantly in cells released synchronously into the cell cycle from a G1 arrest (Ewald et al., 2016). This suggests there are still many regulatory interactions coordinating metabolism and growth with cell cycle progression to be discovered. So far, we do not know which metabolic enzymes are targeted by which signaling pathways to control metabolic fluxes during the cell cycle.

To begin to address cell cycle-dependent regulation of metabolism, we performed a time-resolved proteome and phospho-proteome study through the cell cycle in synchronized yeast cultures. While there have been several phospho-proteomics reports on the budding and fission yeast cell cycle (Archambault et al., 2004; Holt et al., 2009; Carpy et al., 2014; Swaffer et al., 2016; Touati et al., 2018; Touati and Uhlmann, 2018), there are two important factors that make this study unique and complementary to previous work: First, we employed a synchronization strategy that releases cells from a G1 arrest without external perturbations of metabolism such as media switches, temperature shifts, addition of toxic chemical, or physical stress (Ewald et al., 2016; Rosebrock, 2017). Second, nearly all yeast cell cycle studies are performed using cells growing on complex or synthetic complete media, while we grow cells on ethanol minimal medium to force cells to activate a much larger repertoire of biosynthetic pathways. We found that more than two hundred phosphorylation sites on metabolic enzymes and transporters change in abundance during the cell cycle. Our data further suggests that metabolic signaling pathways including PKA, Snf1, and Glc7 are transiently regulated during cell cycle progression. Thus, we provide evidence for multiple layers of phospho-regulation that coordinate metabolism with cell cycle progression.

## Materials and Methods

### Cell Cultivation and Synchronization

Cells were grown in 1% ethanol minimal media (1.7 g yeast nitrogen base, 5 g/L ammonium phosphate, 10 ml ethanol, pH adjusted to 5 with potassium hydroxide) at 30°C and 250 rpm orbital shaking. For cell cycle arrest, strain JE 611c (Ewald et al., 2016) was grown on 10 nM estradiol to an OD of approximately 0.2. Cells were filtered, resuspended in estradiol-free medium, and grown for 15 h. These G1 arrested cells were released by addition of 200 nM estradiol (dissolved at 1 mM in 100% ethanol). Cell cycle release was monitored by manual bud counting (>200 cells per sample) at 60x magnification.

### Sampling, Protein Extraction, and Digestion

Twenty milliliter of cell culture (OD ∼0.6) were sampled into 1.5 volumes of 60% methanol and precooled to −40°C to quench metabolic activity. Cells were spun at 4000 g. The pellets were frozen in liquid nitrogen and then stored at −80°C until further use. Cells were lysed by bead beating in 8M urea, 150 mM NaCl, 5 mM DTT, 50 mM HEPES pH 8 supplemented with 1x Halt^TM^ Protease and Phosphatase Inhibitor Cocktail (Thermo Fisher Scientific). The lysate was centrifuged at 13,200 rpm for 15 min and the supernatant was transferred to fresh test tubes for a second round of centrifugation. Lysates from two parallel samples were combined to increase starting material. This was followed by an alkylation step using 14 mM iodoacetamide for 45 min at room temperature in the dark and the reaction was then quenched with DTT. In order to clean the proteins a methanol-chloroform precipitation was performed and the protein pellet was washed twice with acetone. The pellet was re-suspended with 8M urea in 50 mM HEPES (pH 8) and the total protein concentration was determined using the Pierce^TM^ BCA Protein Assay Kit (Pierce, Rockford, IL, United States). Approximately 4 mg of protein of each sample were diluted to 4 M urea using 50 mM HEPES (pH8) and digested with LysC (1:100) for 4 h at room temperature. Samples were further diluted to 1 M urea using 50 mM HEPES (pH8) and trypsin (Promega, Madison, WI) was added at a ratio of 1:20 enzyme:substrate for 16 h at 37°C. The digestion was quenched with formic acid and the peptides desalted using a Sep-Pak C18 1 cc Vac 50 mg Cartridge (Waters, Milford, MA, United States). Five percent of each sample was used for total proteome analysis and the remaining peptide was used for phosphopeptide enrichment.

### Phosphopeptide Enrichment

TiO_2_ powder was resuspended in 2M lactic acid/50% acetonitrile (binding solution) at a concentration of 25 mg/ml. Peptides were resuspended in 400 μl of binding solution and added to 640 μl of TiO_2_ slurry and incubated for 1 h while shaking. The samples were then spun down at 10,000 rpm for 1 min and the supernatant was removed. The TiO_2_ pellet was washed with binding solution twice and then 0.1% trifluoroacetic acid/50% acetonitrile three times. Phosphopeptides were eluted off TiO_2_ using 50 mM KH_2_PO_4_ (pH 10 adjusted with ammonium hydroxide) twice, acidified with formic acid, and desalted using a Sep-Pak C18 column as above.

### TMT Labeling and High-pH Reversed-Phase Fractionation

The TMT labeling reagents were obtained from Pierce and the labeling was performed according to the manufactures suggested procedure and previously published protocol (Zhang and Elias, 2017). In brief, 100 μg samples were resuspended in 100 μl of 50 mM Na-HEPES and then 30 μl of acetonitrile was added to each sample. A TMT-10plex kit was used and each TMT reagent (0.8 mg per vial) was reconstituted in 40 μl of acetonitrile. 10 μl of the reagent was added to the corresponding sample to incubate for 1 h. To reverse unwanted TMT labeling with tyrosine residues, the reaction was quenched with a final concentration of 0.3% (v/v) hydroxylamine for 15 min at room temperature. Samples were acidified with formic acid to pH 2. In order to assess the labeling efficiency a ratio-check was performed by combining 5 μl of each sample, desalting by StageTip and then analyzing with LC-MS. Based on the result from the ratio-check equal amounts of each individual labeled sample were then combined to deliver an overall equal amount across all channels. The combined peptides were desalted using a Sep-Pak C18 column and then fractionated by high-pH reverse phase fractionation (Yang et al., 2012) using an 84 min gradient (buffer A: 10 mM ammonium formate, pH 10; buffer B: 10 mM ammonium formate, 90% ACN, 10% H_2_O, pH 10) on an Agilent 1200 HPLC (Agilent Technologies, Santa Clara, United States). In total 84 fractions were collected, concatenated, combined into a total of 12 fractions, and then dried down. All fractions were desalted using Sep-Pak C18 column, dried down and resuspended in 0.1% formic acid for LC-MS analysis.

### Mass Spectrometry Analysis

Peptides were separated on a 24 cm reversed phase column (100 μm inner diameter, packed in-house with ReproSil-Pur C18-AQ 3.0 m resin, Dr. Maisch GmbH) over 180 min using a two-step linear gradient with 4–25% buffer B (0.2% (v/v) formic acid in acetonitrile) for 120 min followed by 25–45% buffer B for 15 min at a 400 nL/min flowrate on an Dionex Ultimate 3000 LC-system (Thermo Fisher Scientific, San Jose, CA, United States). Eluted peptides were analyzed with a Fusion Lumos mass spectrometry system (Thermo Fisher Scientific, San Jose, CA, United States). Full MS scans were performed in the Orbitrap in the mass range of 400–1500 m/z and the resolution was set to 120,000. The AGC setting was 4E5 and maximum injection time for FTMS1 was 50 ms. Data dependent mode was set to top speed with duty cycle of 3 s. Precursor ions with charge states 2–7 were selected for fragmentation using collision induced dissociation (CID) with quadrupole isolation, isolation window of 0.7 m/z, normalized collision energy of 35% and activation Q of 0.25. MS2 fragments were analyzed in the ion trap mass analyzer with turbo scan rate and maximum injection time of 50 ms. Ions within a ±10 ppm m/z window around ions selected for MS2 were excluded from further selection for fragmentation for 90 s. Following each MS2 CID, a MS3 higher-energy collisional dissociation (HCD) is performed with synchronous precursor selection enabled (the number of precursors set to 5) and collision energy of 65% (McAlister et al., 2014). HCD fragment ions were detected in the Orbitrap in the scan range of 120–500 m/z with resolution of 60,000, AGC setting of 10,000, and maximum ion time of 120 ms. The mass spectrometry proteomics data have been deposited to the ProteomeXchange Consortium via the PRIDE (Perez-Riverol et al., 2019) partner repository under the dataset identifier PXD015235.

### Data Processing

#### Protein Identification and Quantification

Raw data were searched using SEQUEST in Proteome Discoverer 2.2 against a sequence database of yeast (strain W303, NCBI taxonomy ID 559292, downloaded on July 28, 2016). Trypsin was selected as the enzyme with at most two missed cleavage sites. Precursor mass tolerance was set to ± 10 ppm and fragment mass tolerance was set to ± 0.6 Da. At most three dynamic modifications were allowed per peptide. Carbamidomethylation of cysteine (+57.021 Da) and TMT-labeled N-terminus and lysine (+229.163) were set as static modifications. Oxidation of methionine (+15.995 Da) and acetylation of protein N-terminus (+42.011 Da) were set as variable modifications. For phosphopeptides analysis phosphorylation of Serine, Tyrosine and Threonine (+ 79.967) were also set as differential modifications. Percolator was applied to filter incorrect identifications down to an estimated false discovery rate of 1% for both peptides and proteins. The ptmRS node was used for phosphosite assignment. For quantification, a mass tolerance of ±20 ppm window was applied to the integration of report ions using the “most confident” centroid method and S/N values were reported as reporter abundances. For total proteome analysis, the threshold for average reporter S/N was set to 5, the threshold for co-isolation was set to 30%, and quantification results were rejected for missing channels. The data normalization mode was set to “total peptide amount” and scaling mode was set to “on channels average.”

#### Phosphorylation Site Quantification

For phosphosite analysis, PSMs were filtered to meet the following criteria: The phosphosite position confidence (ptmRS score) was set to >75%; the threshold for average reporter S/N was set to 10; and the threshold for co-isolation was set to 30%. Only PSMs quantified in nine consecutive channels were included (so only the first or last time point were allowed to be zero). After filtering, the channels were normalized to the total intensity. PSMs were summed to unique peptides. Each phosphorylated site was then summed across all peptides containing that site. The quantification of each site was scaled by its mean before averaging the replicates.

### Statistical Analysis

#### Heuristic *p*-Value and Ranking

To avoid any *a priori* assumptions of the shape of the time profiles, we ranked our time courses based on a heuristic *p*-value calculated in the following ways. For each phosphorylation site, we calculated a *p*-value from a *t*-test comparing the average of the first four to the last four time points. Also a regression over all timepoints as independent variables was performed to detect linear trends. Finally, we calculated the *p*-value of linear regressions in time windows of five time points moving across the time series to detect trends which do not span the whole time span. All values were corrected for multiple hypothesis testing with the Holm-Sidak correction. The minimum *p*-value obtained from these tests was then used to rank the phosphorylation sites.

To test whether this ranking separates changing from non-changing sites, we performed k-means clustering (see below) on sets of 1,000 sites from top to bottom rank, see Supplementary Figure 1. Based on the results from this clustering, we empirically decided to use the top third ranking phosphorylation sites for further analysis. For each site in each replicate, the correlation between the protein und phosphosite abundance was calculated. Phosphosites that correlated with Pearson’s R greater than 0.8 in either replicate were removed from downstream phosphorylation analysis. Above procedures were carried out with statsmodels (0.9.0) in Python 3.6.8.

#### K-Means Clustering

K-means clustering was performed using the Matlab 2018b built-in algorithm with 1,000 iterations and 100 replicates. The number of clusters was empirically set to five (see Supplementary Figure 2 for results for 4, 6, and 8 clusters).

#### Principal Component Analysis

A principal component analysis was performed on the normalized abundance data using Perseus 1.6.1.3 (Tyanova et al., 2016).

#### Motif Enrichment

Motif enrichment was performed using the MoMo function (Cheng et al., 2019) on the MEME suite (accessed in May 2019)^1^ (Bailey et al., 2009) with the following settings: motif-x algorithm; background peptides extracted from reference sequence GenBank *Saccharomyces cerevisiae* uid 128; motif width 13; central residues with same modification mass combined; *p*-value threshold was set to 0.0001.

#### GO-Term Analysis

GO-term analysis based on “process” was performed using the Gene Ontology Term Finder on the *Saccharomyces* Genome Database https://yeastgenome.org/goTermFinder.

## Results

**FIGURE 1:**
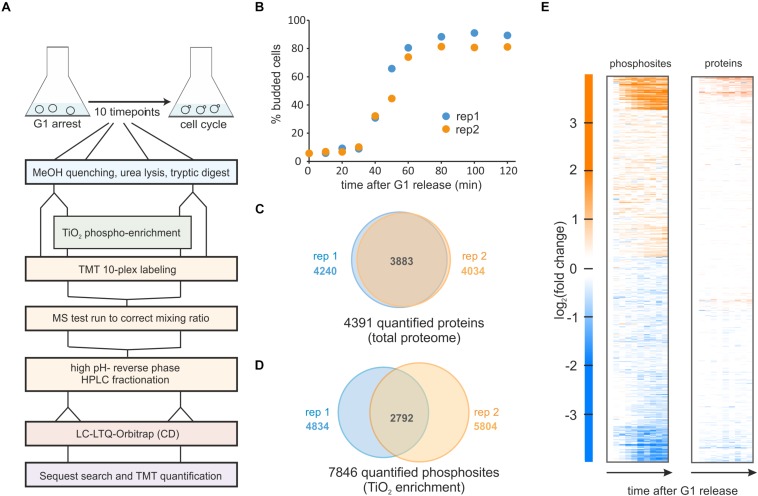
Phospho-proteomics time course of yeast cells released synchronously from G1 on ethanol minimal medium. **(A)** Experimental workflow for sampling, phospho-enrichment, TMT labeling and mass spectrometry analysis. **(B)** Budding index of two replicate cultures released from a G1 arrest. **(C)** Total protein, and **(D)** Phosphorylated sites quantified in the two replicate experiments. **(E)** Heatmap of the averaged replicates (log_2_ fold changes relative to *t* = 0 min) for phosphorylated sites and quantified proteins.

In this study, we wanted to identify mechanisms coordinating metabolism with cell cycle progression. Since both the cell cycle (Morgan, 2007; Enserink and Kolodner, 2010) and metabolic fluxes (Oliveira et al., 2012; Conrad et al., 2014; Chen and Nielsen, 2016) are known to be strongly regulated by phosphorylation, we decided to perform a phospho-proteomics and total proteomics time course of cells progressing through the cell cycle. Specifically, we arrested cells growing on ethanol minimal medium in G1 using our previously described hormone-inducible-cyclin strains (Ewald et al., 2016). These cells lack endogenous G1 cyclins (*cln1Δcln2cln3Δ*) and have an exogenous copy of *CLN1* that is expressed from an estradiol-inducible promoter (*LexApr-CLN1*) (Ottoz et al., 2014). Importantly, this strain can be released from a G1 arrest by adding 200 nM estradiol, which induces G1 cyclin expression reproducibly and highly synchronously without any other detectable cellular perturbations (see Ewald et al., 2016, Supplementary Material for a detailed characterization of the strain). Avoiding perturbations such as media changes, physical or temperature stress during the synchronous release is crucial when aiming to study metabolism, because many metabolic pathways are regulated in response to stress (Gasch and Werner-Washburne, 2002; Brauer et al., 2008). With this hormone-inducible strain, we performed two replicate experiments which showed very similar and highly synchronous budding profiles (Figures 1A,B). We note that we present data for the first 2 h after the G1 release, which corresponds to most cells being in early mitosis and is before cells lose synchrony (Ewald et al., 2016).

From our two cell cycle synchronized cultures, we sampled ten time points from each replicate. Cells were lysed and proteins were digested with trypsin and lysC. Approximately 5% of each sample was removed for total proteome analysis and from the remainder phosphopeptides were enriched with TiO_2__._ Both total proteome and enriched samples were labeled with the TMT-10 plex (Figure 1A and section Materials and Methods).

In our total proteome cell cycle time course, we quantified over 4,000 proteins, with more than 90% overlap between the replicates (Figure 1C and Supplementary Table 1). Using an MS3 approach (25) and stringent quality criteria (see section Materials and Methods) we quantified a total of 9,267 unique phosphopeptides across all time points. This resulted in almost 8,000 quantified phosphorylation sites with approximately half of these quantified in both replicates (Figure 1D and Supplementary Table 2). As reported in previous studies (Godfrey et al., 2017; Touati et al., 2018; Touati and Uhlmann, 2018) the overall changes in the proteome through the cell cycle are small. In contrast, approximately one third of all phospho-sites change in abundance at least twofold during the cell cycle suggesting cell cycle-dependent phosphorylation of these sites (Figure 1E).

**FIGURE 2:**
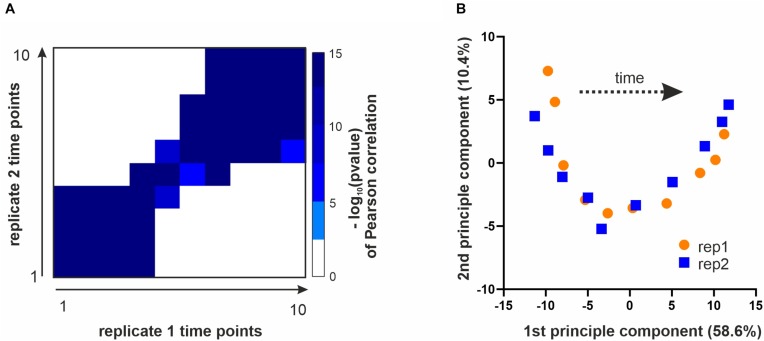
Data overview and quality controls. **(A)** All time points of replicate 1 were correlated with all time points from replicate 2 (based on top 3rd ranking phosphosites, see section Materials and Methods). Shown is a heatmap of the –log_10_(*p*-value) of a Pearson correlation for all time points of one replicate with those of the other replicate. **(B)** Principle component analysis performed with the top 3rd of the identified phosphosites. Plotted are the ten time points of each replicate projected onto the first two principle components.

Next, we sought to identify which phosphorylation sites were regulated during the cell cycle and test the quality and reproducibility of our phosphoproteome data. We first ranked the time profiles of all phosphorylation sites based on a heuristic *p*-value of change across the cell cycle (see section Materials and Methods). We then removed sites from further analysis that strongly correlated with total protein abundance, since these are unlikely to be regulated mainly by phosphorylation. We used the top third of the sites based on our ranking for further analysis (Supplementary Figure 1). To test the quality and reproducibility of our data, we correlated all ten time points of replicate 1 with all ten time points of replicate 2. Samples from corresponding times after release correlated well with *p*-values (Pearson correlation) of 10^–15^ or less for each of the ten time points (Figure 2A). As expected, neighboring time points show a higher degree of correlation than more distant data points. Moreover, a principle component analysis (PCA) separated the samples according to the time they were taken along the first component, and replicate samples were positioned near each other in the first two PCA components (Figure 2B), an indication of the accuracy of the acquired data. To test if our data and ranking capture known cell cycle regulation, we analyzed the GO-term categories for the top-ranking phospho-sites. Indeed, 36 of the top 100 ranking phosphoproteins are annotated to the GO-term “cell cycle” (3.3-fold enrichment over genome, *p* < 10^–7^) and 63 of these proteins are annotated to the more general category “biological regulation” (2.1-fold enrichment over genome, *p* < 10^–8^).

**FIGURE 3:**
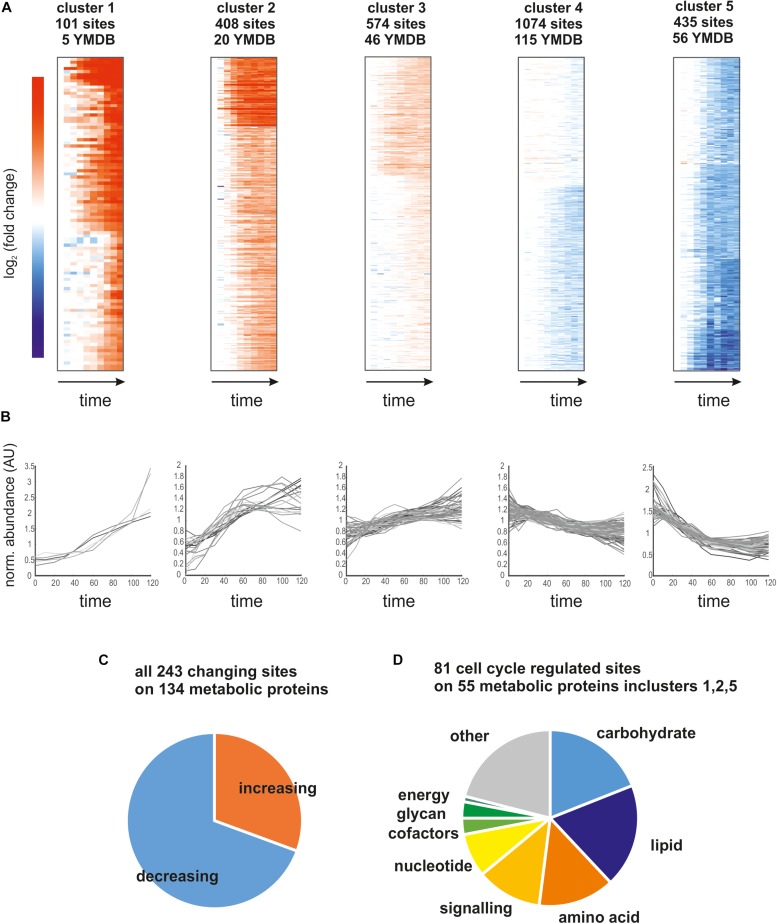
**(A)** Heatmaps of the five identified clusters based on log_2_ (fold change) relative to *t* = 0 min. We report the number of sites contributing to the cluster and how many of those map to proteins in the yeast metabolome database (YMDB). **(B)** Time course of phosphosite abundance for all sites on a YMDB protein in the corresponding cluster. **(C)** Pie chart reporting the fraction of phosphosites on YMDB metabolic proteins whose abundance is increasing or decreasing through the cell cycle. **(D)** Pie chart reporting the pathway assignments of the phosphorylation sites whose abundance changes the most through the cell cycle.

Having established the quality of our phosphoproteomics time course, we next investigated which metabolic enzymes were dynamically phosphorylated and possibly regulated. To analyze the trends in the data set and how they relate to metabolism, we clustered the top-ranking sites using k-means clustering into five distinct clusters (Figures 3A,B; four, six, and eight clusters give qualitatively similar results as shown in Supplementary Figure 2). For each cluster, we analyzed which of the phosphorylation sites were annotated to proteins listed in the yeast metabolome database (Ramirez-Gaona et al., 2017; Figure 3A). Proteins related to metabolism were found in every cluster, and, in total 243 sites on 134 metabolic proteins were changing (Figure 3B). Interestingly, more sites on these metabolic proteins were dephosphorylated than phosphorylated (Figure 3C). To determine which metabolic pathways were most likely affected by phospho-regulation, we sorted the 81 most dynamic sites on metabolic proteins from clusters 1, 2, and 5 (the clusters with the largest average fold-change) into KEGG categories (Figure 3D). All major metabolic pathways were represented and there was no particular category enriched relative to the whole dataset. In line with our previous metabolomics data showing that over half of ∼500 measured metabolites change throughout the cell cycle (Ewald et al., 2016), these phosphoproteomics data suggest that global adaptations across metabolism are occurring during the cell cycle and are at least in part regulated by phosphorylation.

**FIGURE 4:**
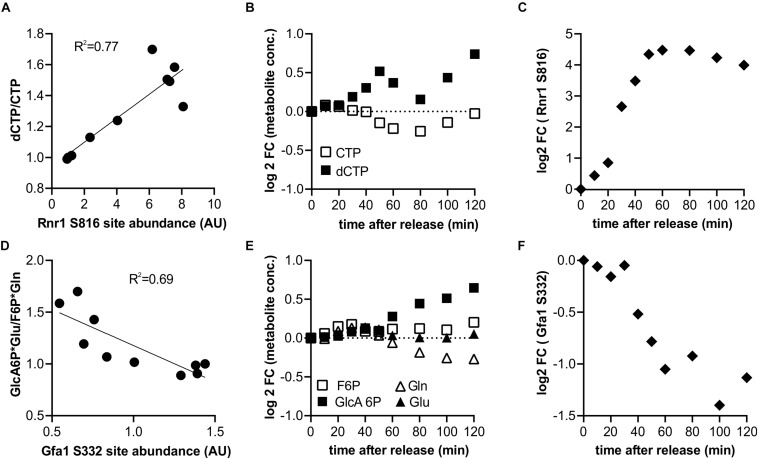
Identification of putative potential flux controlling phosphorylation sites based on the product to substrate ratio from published metabolomics data (6). **(A–C)** Example of a putative activating phosphorylation site showing the correlation of the serine 816 on ribonucleoreductase 1 (Rnr1) with the dCTP to CTP ratio, and the corresponding cell cycle time courses. **(D–F)** Example of a putative inhibiting phosphorylation site showing anti-correlation of serine 332 of Glutamine-fructose-6-phosphate amidotransferase (Gfa1) with the ratio of its products and substrates.

We next wanted to determine which of the measured changes in enzyme phosphorylation may directly contribute to changes in metabolic activity. As a rough approximation of metabolic activity we use the product-to-substrate ratios from our previous metabolomics data set (Ewald et al., 2016) which was obtained with the same strain under identical growth conditions A change in the product-to-substrate ratio indicates a change in the kinetics of the reaction. For 174 sites on 82 proteins in our data set we had at least one substrate and one product (not including cofactors) for the reaction catalyzed by the phosphorylated enzyme. For each of these reactions we correlated the phospho-site abundance with the product-to-substrate ratio (Supplementary Table 3). We found 19 sites on 15 enzymes with an R^2^ of the correlation greater than 0.5 (Supplementary Figure 3). One example is an enzyme well known to be tightly coordinated with the cell cycle through multiple mechanisms: the ribonucleotide-reductase complex, which catalyzes the conversion of NTPs to dNTPs (Lowdon and Vitols, 1973; Sanvisens et al., 2013). The CDK consensus site S816 on Rnr1 correlates well with the ratio of dCTP to CTP (We note that cytosine nucleotides were chosen as example since they have unique masses in our metabolome data set and they do not participate in as many other reactions as adenylate or guanylate nucleotides) (Figures 4A–C). It therefore seems likely that Rnr1 S816 contributes to activating enzyme activity; this could either be direct or indirect by supporting known regulatory mechanisms such as localization and oligomerization (Sanvisens et al., 2013). Additionally, Rnr1 is also transcriptionally upregulated, but the increase in phosphorylation on S816 greatly exceeds the increase in total protein (Supplementary Figure 4). A second example is glutamine-fructose-6-phosphate amidotransferase (Gfa1), which catalyzes the first step in the chitin pathway necessary for cell wall synthesis. The site S332 on this Gfa1 is dephosphorylated during the cell cycle which anti-correlates with the product to substrate ratio (Figures 4D–F). We therefore suggest that this is an inhibitory phosphorylation which is being released during the cell cycle to increase chitin synthesis for surface expansion and cytokinesis. Whether this dephosphorylation is directly regulated by the cell cycle machinery or whether it is a secondary effect downstream of other metabolic changes (such as trehalose and glycogen utilization; Ewald et al., 2016; Zhao et al., 2016) remains to be investigated. The resulting slopes and R^2^ of all correlations that could be determined based on the two datasets are reported in Supplementary Table 3.

**FIGURE 5:**
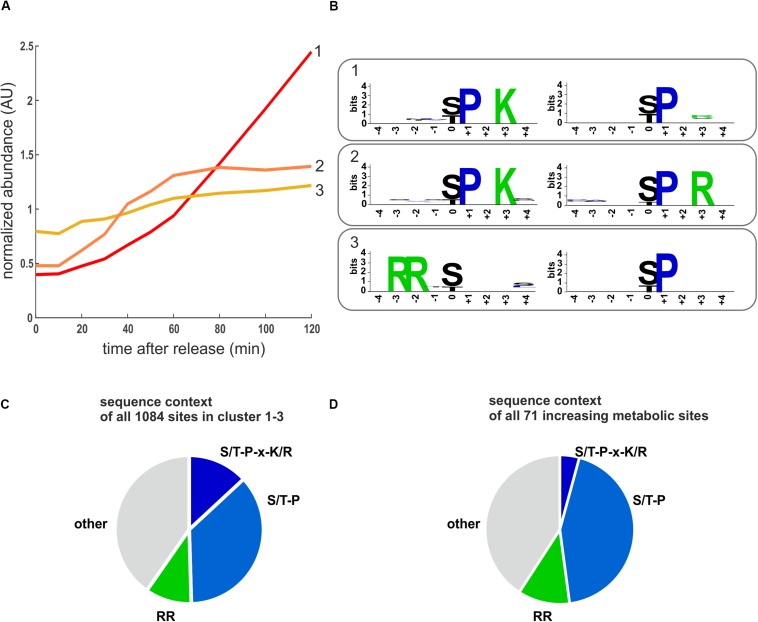
Motifs enriched in increasing phosphorylation sites. **(A)** Cluster averages of three increasing clusters identified by k-means clustering. **(B)** Enriched motifs identified in the increasing clusters using the motif-X algorithm. The two most enriched motifs for each cluster are shown (*p* < 10^–6^), the complete motif analysis for all clusters is reported in Supplementary Table 4. **(C)** Pie chart depicting the sequence context of all sites in the cell cycle increasing clusters 1–3. **(D)** Pie chart depicting the sequence context of the cell cycle increasing phosphosites on metabolic proteins. RR denotes motifs potentially recognized by PKA including RRxS, RRxxS, and RxRxS. S/T-P-x-K/R is the optimal CDK consensus site.

To investigate which kinases contribute most to increasing phosphorylation in metabolic and all other proteins, we performed an unbiased motif analysis using the motif-x algorithm (Schwartz and Gygi, 2005) implemented on the Meme-suite (Bailey et al., 2009; Cheng et al., 2019). Not surprisingly, the two clusters corresponding to phosphorylation sites increasing early and late through the cell cycle were highly enriched for CDK consensus sites (S/T-P-X-K/R) and minimal CDK sites (S/T-P) sites (Figures 5A,B and Supplementary Table 4). However, the most enriched motif in the gradually increasing cluster 3 was RRxS/T and not proline-directed (Figure 5B and Supplementary Table 4). This motif is the consensus sequence associated with the protein kinase A (PKA) and some other metabolic kinases (Ptacek et al., 2005; Mok et al., 2010). We note that RRxS/T is also a motif for the cell cycle regulator aurora kinase (Ipl1) (Mok et al., 2010). However, aurora is thought to be mainly active during G2/M (Ruchaud et al., 2007; Krenn and Musacchio, 2015). Consistently, we find increasing phosphorylation on Ipl1 and its regulatory subunit Sli15 during later time points in our data set (Supplementary Figure 5), making it unlikely that it is the major contributor to phosphorylation in cluster 3. In clusters 1–3, which contained all sites increasingly phosphorylated through the cell cycle, almost half were proline directed and 15% were arginine directed (putative PKA targets) (Figure 5C). When we were only examining phosphorylation sites on metabolic proteins, we obtained a similar distribution (Figure 5D).

**FIGURE 6:**
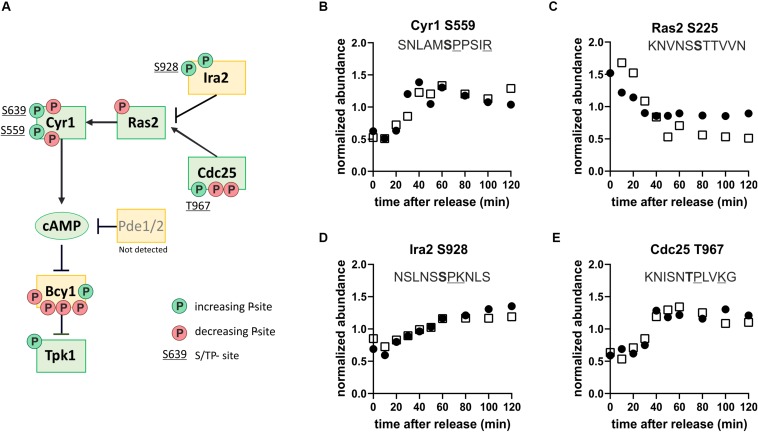
The protein kinase A pathway is phospho-regulated through the cell cycle. **(A)** Map of the Ras-branch of the PKA pathway. Circles indicate sites whose phosphorylation increases (green, clusters 1–3) or decreases (red, clusters 4–5) through the cell cycle. Only sites found in both replicates are reported. S/TP sites, possibly phosphorylated by cyclin-dependent kinases, are denoted by their residue numbers adjacent to the phosphorylation site. **(B–E)** Examples of dynamic phosphorylation of sites on different upstream regulators of PKA through the cell cycle. Residues associated with consensus cyclin-dependent kinase sites are underlined and the phosphorylated residue is shown in bold. Black and white symbols denote the two replicate time courses.

That we identified consensus PKA phosphorylation sites as being dynamic through the cell cycle is interesting because PKA kinase is a sensor of nutrients (mainly glucose) and environmental stresses. PKA promotes cell growth and glucose repression and inhibits several stress responses (Broach, 2012; Conrad et al., 2014). Since we did not change the nutrient or stress conditions of our yeast cultures, we wanted to further investigate how putative PKA target sites could be increasingly phosphorylated during cell cycle progression. We noticed that several regulators upstream of PKA seemed to be phospho-regulated during cell cycle progression, with several phosphorylation sites either increasingly or decreasingly phosphorylated through the cell cycle (Figure 6). Many of the increasingly phosphorylated sites were proline directed (Figures 6B,D,E) and were similar to CDK consensus sites. This suggests that the Ras-branch of the PKA pathway could be activated by the cell cycle machinery to control downstream processes in metabolism and growth.

In addition to examining the sites increasingly phosphorylated through the cell cycle, we also wanted to investigate the sites being dephosphorylated through the cell cycle because they could be equally important. Dephosphorylation during the cell cycle is mainly discussed in the context of phosphatases counteracting CDK phosphorylation when cells go through mitosis (Mochida and Hunt, 2012; Rogers et al., 2016; Kataria et al., 2018) and in early G1 (Godfrey et al., 2017). In our experiment, we noticed that there are at least as many dephosphorylation events as phosphorylation events during the G1/S transition and S-phase, which are cell cycle transitions typically associated with increasing kinase activity. For metabolic proteins, twice as many sites were dephosphorylated through G1 to S as phosphorylated.

**FIGURE 7:**
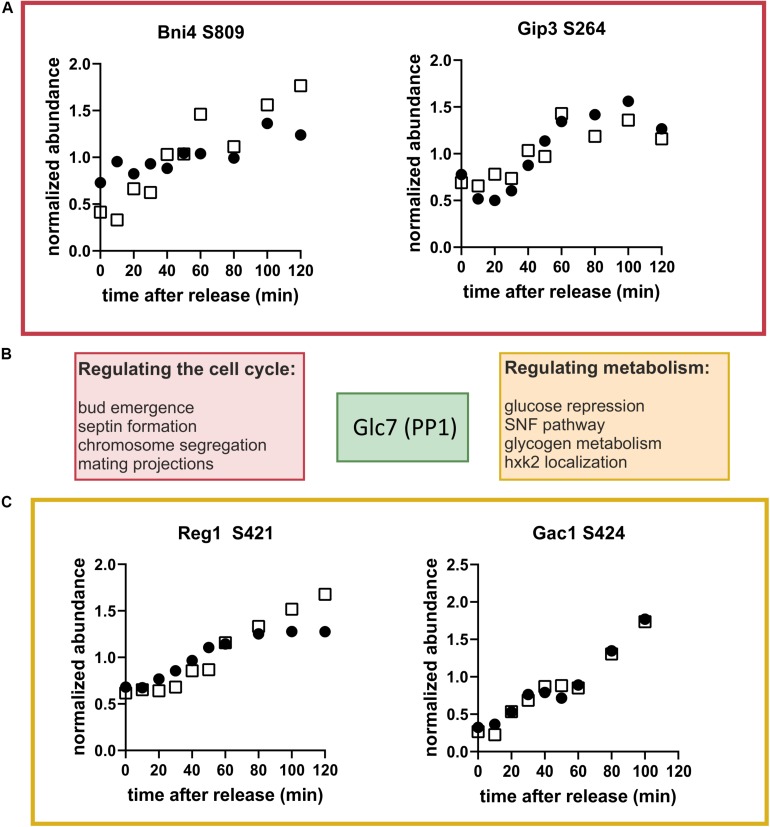
Glc7 (PP1) may regulate metabolism through the cell cycle. **(A)** Cell cycle-dependent phosphorylation of the Glc7 subunits, Bni4 and Gip3, which are known to contribute to cell cycle regulation. **(B)** Schematic showing regulatory subunits of the phosphatase Glc7 and their annotated functions. **(C)** Cell cycle time courses of phosphorylation of the Glc7 subunits Reg1 and Gac1, which are known to contribute to metabolic regulation. Time courses from both replicates are shown. Black and white symbols denote the two replicate time courses.

The prevalence of dephosphorylation through the cell cycle led us to wonder which phosphatases could be contributing or whether sites of specific kinases where being targeted. Motif enrichment analysis of clusters 4 and 5 (see Supplementary Table 4) did not direct us toward a specific kinase or phosphatase. However, we noticed that one of the top-ranking phosphorylation sites in our list was on Reg1, a regulatory subunit of the phosphatase Glc7 of the well-conserved PP1 family (Verbinnen et al., 2017). Glc7 has many targets and important functions in the cell cycle and in carbon metabolism (Cannon, 2010). Glc7 obtains its specific activity through interactions with regulatory subunits like Reg1 (Figure 7B) and has little specificity on its own. It does not seem to be regulated in abundance or in its phosphorylation state during the cell cycle (Supplementary Tables 1, 2). Motivated by the identification of Reg1 as a dynamically phosphorylated protein, we searched our list of high-ranking phosphorylation sites for other Glc7 subunits. We found regulatory subunits that are known to regulate cell cycle functions including Bni4, which regulates bud neck and septum assembly, and Gip3, which regulates chromosome segregation (Figure 7A). Additionally, several of the subunits involved in regulating metabolism including Reg1 (glucose repression) and Gac1 (glycogen metabolism) were dynamically phosphorylated (Figure 7C). Although we did not find any annotated functions to these specific sites, it is tempting to speculate that these phosphorylation sites impact either binding of its targets or binding of the regulatory subunit to the catalytic subunit.

**FIGURE 8:**
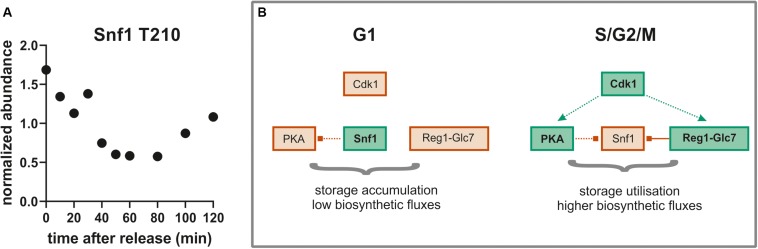
**(A)** The well conserved activating site T210 on Snf1 is dephosphorylated during the G1-S- transition (average of both replicates). **(B)** Model for global metabolic regulation during cell cycle progression on ethanol minimal medium. Red, low activity; green, higher activity; dotted lines, indirect or putative regulatory interactions; solid line, direct regulatory interaction.

To further investigate the idea that the cell division cycle drives changes in Glc7 phosphatase activity, we searched for known Glc7-Reg1 targets among our list of dephosphorylated sites. One of the most prominent targets of Glc7-Reg1 is the kinase Snf1 (homolog of mammalian AMPK; Hardie, 2011). Snf1 is activated in the absence of glucose by phosphorylation on site T210 (Conrad et al., 2014). This activating phosphorylation is counter-acted by dephosphorylation by Reg1-Glc7 (Tu and Carlson, 1995). Consistent with our model, we find that Snf1 T210 is decreasing in abundance during the G1/S transition and seems to recover later in the cycle (Figure 8A). This was surprising given that Snf1 normally responds to changes in external glucose, which was constantly absent throughout our experiment. In response to glucose limitation, Snf1 regulates several aspects of carbon metabolism including the deactivation of the transcription factor Mig1. Mig1 is phosphorylated by Snf1 on at least four sites in its nuclear localization sequence and at least some of these sites are also reported to be dephosphorylated by Reg1-Glc7 (Smith et al., 1999). We therefore wondered whether Mig1 was also phospho-regulated during the cell cycle. We found one site S302, which closely follows the pattern of Snf1 dephosphorylation (Supplementary Figure 6A). While this site has not been specifically reported to be either a Snf1 or Reg1 target, it lies right between two Snf1 sites within the regulatory domain of Mig1 (Supplementary Figure 5C). Another site, T371, also lies within the Mig1 regulatory domain and is increasingly phosphorylated through the cell cycle (Supplementary Figure 6B). Interestingly, this site contains a proline in + 1, which may point to phosphorylation by CDK1 as suggested by earlier studies (Holt et al., 2009; Zhao et al., 2016). A GFP-tagged Mig1 did not change localization during the cell cycle under our growth conditions (Supplementary Figure 7), suggesting these phosphorylation sites regulate Mig1 in a localization-independent way.

## Discussion

The aim of this study was to identify mechanisms coordinating metabolism and growth with the cell division cycle in budding yeast. Since both metabolism (Conrad et al., 2014; Chen and Nielsen, 2016) and the cell cycle (Morgan, 2008; Enserink and Kolodner, 2010) are extensively phospho-regulated, we performed a phosphoproteomics time-course of cells released from a G1 arrest. In contrast to previous phospho-proteomics studies, our main focus was to explore the phospho-regulation of metabolism through the cell cycle. We therefore took extreme care to employ a synchronization strategy that would not lead to metabolic alterations through media changes or stress responses. To achieve this, our study was conducted with prototrophic strains growing on ethanol minimal medium, where cells grow slowly and need to activate their full biosynthetic potential. Our novel high quality dataset is therefore complementary to other phosphoproteomics data sets on the yeast cell cycle (Archambault et al., 2004; Holt et al., 2009; Touati et al., 2018; Touati and Uhlmann, 2018).

In summary, we found over 200 sites on metabolic enzymes that were either increasingly phosphorylated or dephosphorylated throughout the cell cycle. In agreement with our previous metabolomics study (Ewald et al., 2016), many different metabolic pathways were affected including carbohydrate, lipid, amino acid and nucleotide metabolism. While most of these sites still need to be functionally validated, the sheer number of phosphorylated or dephosphorylated sites suggests that phosphorylation contributes significantly to tailoring metabolic fluxes to the specific requirements of different cell cycle phases.

The identification of large-scale changes in phospho-isoforms through the cell division cycle raised the question as to which signaling pathways were responsible. We and others previously showed that the CDK directly regulates the activity of several metabolic enzymes such as the trehalase Nth1 (Ewald et al., 2016; Zhao et al., 2016) and the lipase Tgl4 (Kurat et al., 2009). This is unlikely to represent the full extent of metabolic regulation by CDK because previous work on rich media identified several other metabolic enzymes that were likely phosphorylated by CDK (Ubersax et al., 2003; Holt et al., 2009; Zhao et al., 2016). Using our minimal media conditions, we further expand the list of putative direct CDK targets in metabolism. However, the data also suggest that a direct regulation of enzymes by the cell cycle-dependent increase in proline directed CDK activity is not the main driver of adjusting metabolic fluxes, since many enzymes get dephosphorylated rather than phosphorylated, and only a minority of all phosphorylated sites are proline directed. We therefore suggest that a lot of the cell cycle-dependent phospho-regulation controlling metabolic fluxes is not directly through CDK activity, but entails additional pathways.

One such additional pathway could be the protein-kinase A signaling pathway. Our data suggests that the PKA pathway is cell cycle regulated and in turn contributes to cell cycle-dependent phosphorylation of downstream pathways. Two independent observations lead to this conclusion. First, the PKA consensus motif RRxS was found as highly enriched in one of the clusters of sites being increasingly phosphorylated through the cell cycle and 15% of all increasing sites were arginine directed. Second, many of the upstream regulators in the Ras-branch of the PKA pathway change in phosphorylation state during the early cell cycle. These sites have yet to be functionally annotated, but we speculate that at least some of these phosphorylation sites modulate activity of the proteins or interaction between the signaling partners. Many of these phosphorylation sites are proline directed, raising the possibility that CDK itself activates PKA signaling. Alternatively, MAP kinases related to cell cycle progression such as Kss1 or Slt2, which are also proline directed, could be involved in phosphorylation. Either way, regulation of PKA by cell cycle kinases would provide a mechanistic explanation of the spikes in cyclic-AMP concentrations at the G1/S and G2/M transitions observed previously (Muller et al., 2003). Since PKA has been reported to regulate CDK activity at the G1/S transition (Tokiwa et al., 1994; Amigoni et al., 2015; Ewald, 2018), it is likely that the interplay between CDK and PKA is at the nexus coordinating metabolism, growth and division with nutrient supply.

While the putative PKA and CDK sites we identified are increasingly phosphorylated through the cell cycle, for many of the sites we identified the opposite is true. We were surprised at the large amount of dephosphorylation we observed as cells pass the G1/S transition. Many of these targets were metabolic enzymes. This large-scale dephosphorylation may be in part due to changing activity of the phosphatase Glc7/PP1 together with its subunits associated with metabolism such as Reg1 and Gac1. Reg1 also targets and inactivates another important metabolic signaling pathway such as the Snf1 kinase, a member of the highly conserved AMPK family. Snf1 has a well characterized activating site T210 that is phosphorylated by upstream sugar sensing kinases and is dephosphorylated by Reg1. In both of our replicates, Snf1 T210 is dephosphorylated at the G1/S transition and re-phosphorylated as cells progress into mitosis consistent with the hypothesis that changing phosphatase activity may drive large-scale dephosphorylation through G1/S.

The dephosphorylation of Snf1 through G1/S may be important because when Snf1 is activated (like AMPK in mammals) it acts as a “brake pedal” slowing growth and energy consuming processes (Ghillebert et al., 2011; Coccetti et al., 2018). Thus, Snf1 inactivates many processes typically activated by PKA (Nicastro et al., 2015). During entry into the cell cycle at G1/S, phosphoregulation may shift the balance between PKA and Snf1 to enhance growth promoting pathways and rewire metabolism to turn storage compounds such as trehalose, glycogen, or lipid droplets into macromolecules that support cell cycle progression (Figure 8B). This fine-tuned metabolic regulation likely does not matter much under the nutrient rich growth conditions (SCD, YPD) that most cell cycle studies are conducted in but may be crucial in nutrient poor environments such as the ethanol minimal medium we used in this work. Additionally, Snf1 was recently shown to play an important role in spindle alignment and regulation of the meta-to-anaphase transition in a Kar9 dependent manner (Tripodi et al., 2018). This would explain why Snf1 is re-phosphorylated during later cell cycle stages. These observations suggest that there are multiple interactions between the Snf1 pathway and the cell cycle machinery that warrant further exploration.

Taken together, this and other work over the last decade (Kurat et al., 2009; Bryan et al., 2010; Goranov and Amon, 2010; Ewald et al., 2016; Zhao et al., 2016), shows that we need to revise the text book model that cell growth drives the cell cycle but not vice versa. Yeast physiology is likely determined by extensive cross talk between global regulators of metabolism, signaling pathways promoting growth, and the cell cycle control machinery (Papagiannakis et al., 2017; Ewald, 2018; Ozsezen et al., 2019). More broadly, it seems safe to assume that all eukaryotes have extensive, multidirectional signaling mechanisms to coordinate metabolism, growth and the cell division cycle, given the many recent reports on the role of metabolism in proliferating tissues including cancer-, immune-, or stem cells (Vander Heiden and DeBerardinis, 2017; Corbet, 2018; Pearce and Pearce, 2018; Zhang et al., 2018; Dahan et al., 2019; Vaupel et al., 2019). We anticipate that over the coming decade this picture of interlinked metabolic and cell cycle control will be fleshed out as a broad array of post-translational modifications and allosteric interactions mediating cross-talk between metabolism and the cell division cycle are identified in model organisms and in humans.

## Data Availability Statement

Processed data have been included as Supplementary Tables. The raw mass spectrometry proteomics data have been deposited to the ProteomeXchange Consortium via the PRIDE (Perez-Riverol et al., 2019) partner repository under the dataset identifier PXD015235.

## Author Contributions

JCE, JS, and JEE conceived and designed the study. JCE, LZ, and FS performed the experiments. LZ and JEE performed mass spectrometry and raw data analysis. SW and JCE performed statistical analysis. OK advised on data analysis. JCE and JS wrote the manuscript. All authors read and approved the manuscript.

## Conflict of Interest

The authors declare that the research was conducted in the absence of any commercial or financial relationships that could be construed as a potential conflict of interest.

## Footnotes

1 http://meme-suite.org/
